# Medical Communications of the Massachusetts Medical Society

**Published:** 1860-08

**Authors:** 


Medical Communications of the Massachusetts Medical Society. With an Appendix, containing the Proceedings of the Councillors, and of the Society. Vol. ix.—No. vi.—1860.

To the courtesy of the President, Dr. John Homans, of Boston, we are indebted for a copy of the above. The bulk of the present number, which is gotten up with its usual degree of typographical elegance, is occupied by the Annual Address—*Currents and Counter-Currents in Medical Science*—of Dr. O. W. Holmes. The remainder of its contents embrace the List of Deceased Fellows, Obituaries, Proceedings of the Committees, Board of Trial, Proceedings of the Society, Treasurer's Report, Officers of the Society, Officers of the District Medical Societies, List of Fellows admitted since 1854, Index, etc.,—valuable, all, for reference, but not, in the main, of general interest, except to the local reader. As exceptions, however, we note the appointment of a committee to inquire into the expediency of the Society's co-operating with the Medical Profession of Great Britain, which is about to erect a monument in Westminster Abbey, to the memory of John Hunter; also the appointment of a committee to urge upon the Legislature the establishment of a Scientific Commission, to investigate the Cattle Disease.

With its characteristic good sense, the Society, at its May meeting, re-elected its former excellent President, Dr. John Homans; its indefatigable Secretary, Dr. John B. Alley, and other of its former officers. F. R.

